# Supplementary material for: Increased variability in Greenland Ice Sheet runoff from satellite observations
Source: Nat Commun. 2021 Nov 1;12:6069. doi: 10.1038/s41467-021-26229-4 (PMC8560907; doi:10.1038/s41467-021-26229-4)
Supplement: Supplementary file 1 — Supplementary information [file 41467_2021_26229_MOESM1_ESM.pdf]

Supplementary information for:

Increased variability in Greenland Ice Sheet runoff from satellite observations

Thomas Slater[1]*, Andrew Shepherd[1], Malcolm McMillan[2], Amber Leeson[2], Lin Gilbert[3], Alan Muir[3,4], Peter Kuipers Munneke[5], Brice Noël[5], Xavier Fettweis[6], Michiel van den Broeke[5], Kate Briggs[1]

[1]Centre for Polar Observation and Modelling, School of Earth and Environment, University of Leeds, Leeds, UK

[2]Lancaster Environment Centre, Lancaster University, Lancaster, UK

[3]Mullard Space Science Laboratory, Department of Space & Climate Physics, University College London, London, UK

[4]Centre for Polar Observation and Modelling, Department of Earth Sciences, University College London, London, UK

[5]Institute for Marine and Atmospheric research Utrecht, Utrecht University, Utrecht, the Netherlands

[6]SPHERES Research Unit, Department of Geography, University of Liège, Liège, Belgium

Corresponding author: t.slater1@leeds.ac.uk

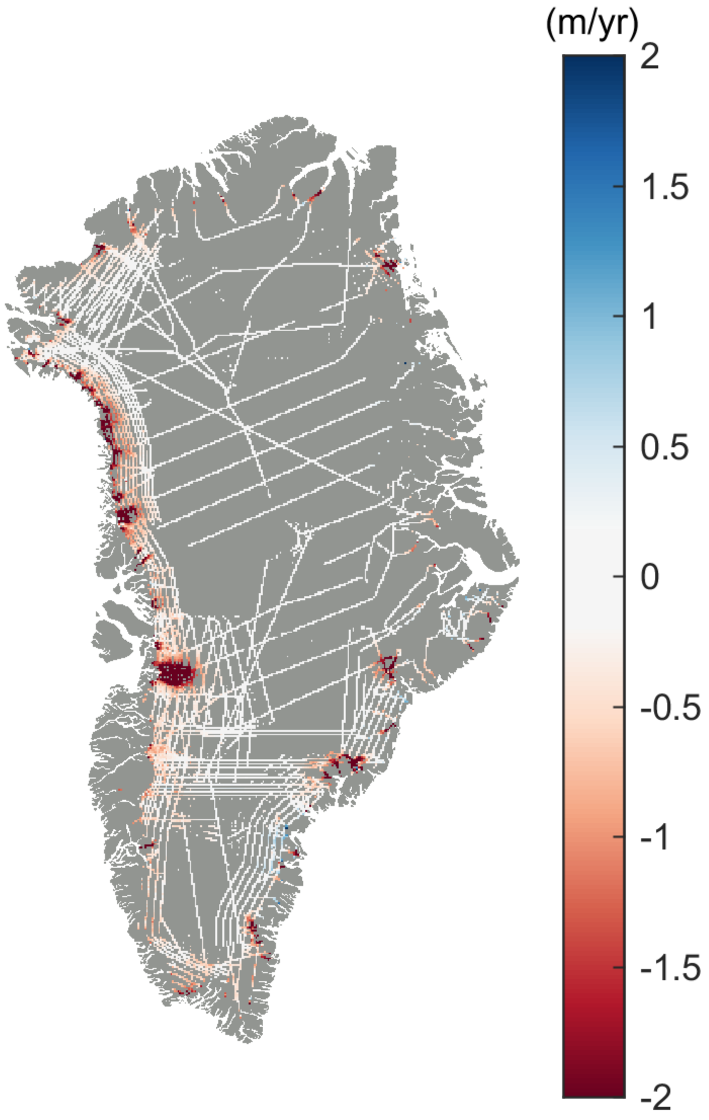

Supplementary Fig. 1. Rates of elevation change derived from Operation Icebridge airborne laser altimetry[32], acquired between 2011 and 2019, used to validate Cryosat-2 elevation change rates (Fig. 1).

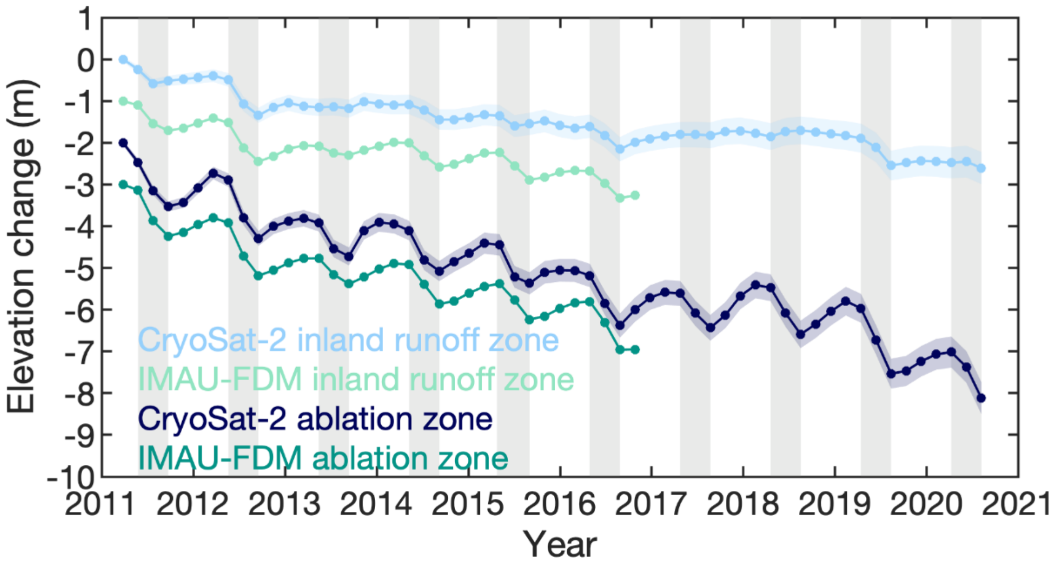

Supplementary Fig. 2. Surface height evolution in the ablation zone (dark) and the area of the runoff zone inland from the ablation zone (light) (Fig. 2) derived from CryoSat-2 altimetry (blue) and the Institute for Marine and Atmospheric Research Utrecht Firn Densification Model (IMAU-FDM) (green) between January 2011 and October 2020. Time-series have been successively offset by 1 m for ease of viewing.

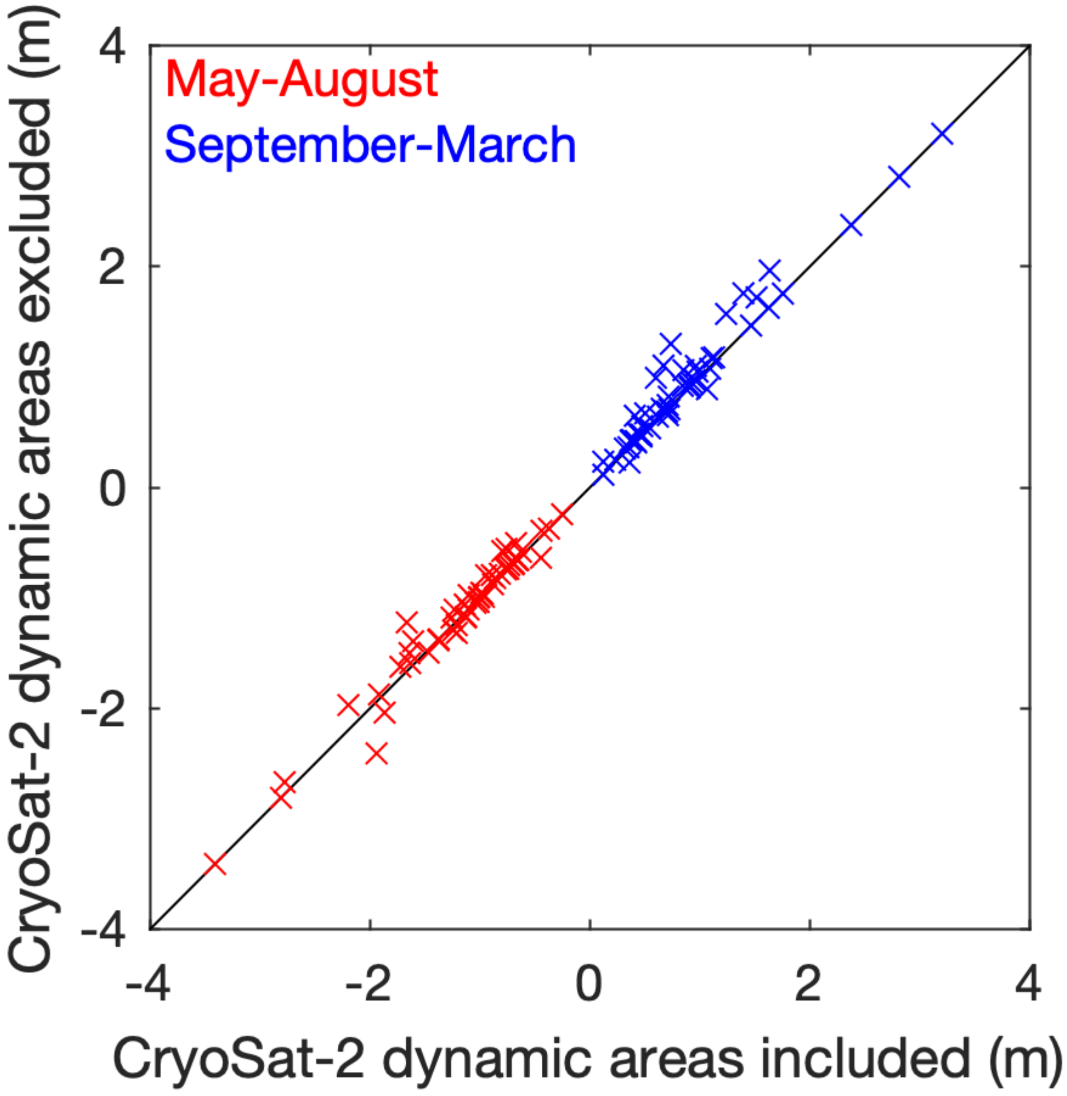

Supplementary Fig. 3. Comparison of summer (red) and winter (blue) elevation changes in the ice sheet ablation zone between 2011 and 2020, including and excluding regions of long-term dynamic imbalance (Fig. 1).

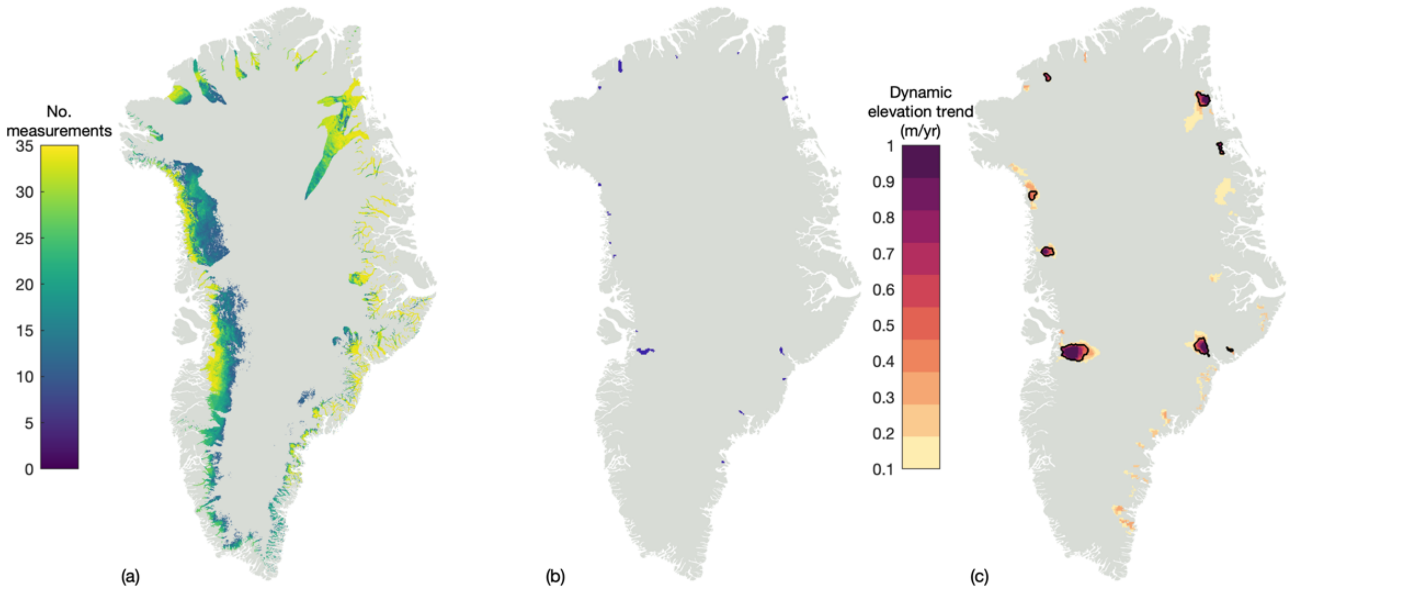

Supplementary Fig. 4. Identification of areas of ice dynamical imbalance through a combination of ice velocity data acquired between 1985 and 2018 from optical imagery[48,49], and dynamic elevation trends determined from satellite altimetry and the Institute for Marine and Atmospheric Research Utrecht Firn Densification Model (2011-2017). (a) Number of available velocity measurements in areas flowing faster than 50 m/yr. (b) Areas of ice dynamical imbalance (blue) identified using ice velocity data. (c) Areas of dynamical imbalance classified where dynamic elevation trends exceed thresholds from 0.1 m/yr to 1.0 m/yr. The contoured area (black) highlights the threshold used in this study, based upon the dynamic elevation trend in areas identified in (b).

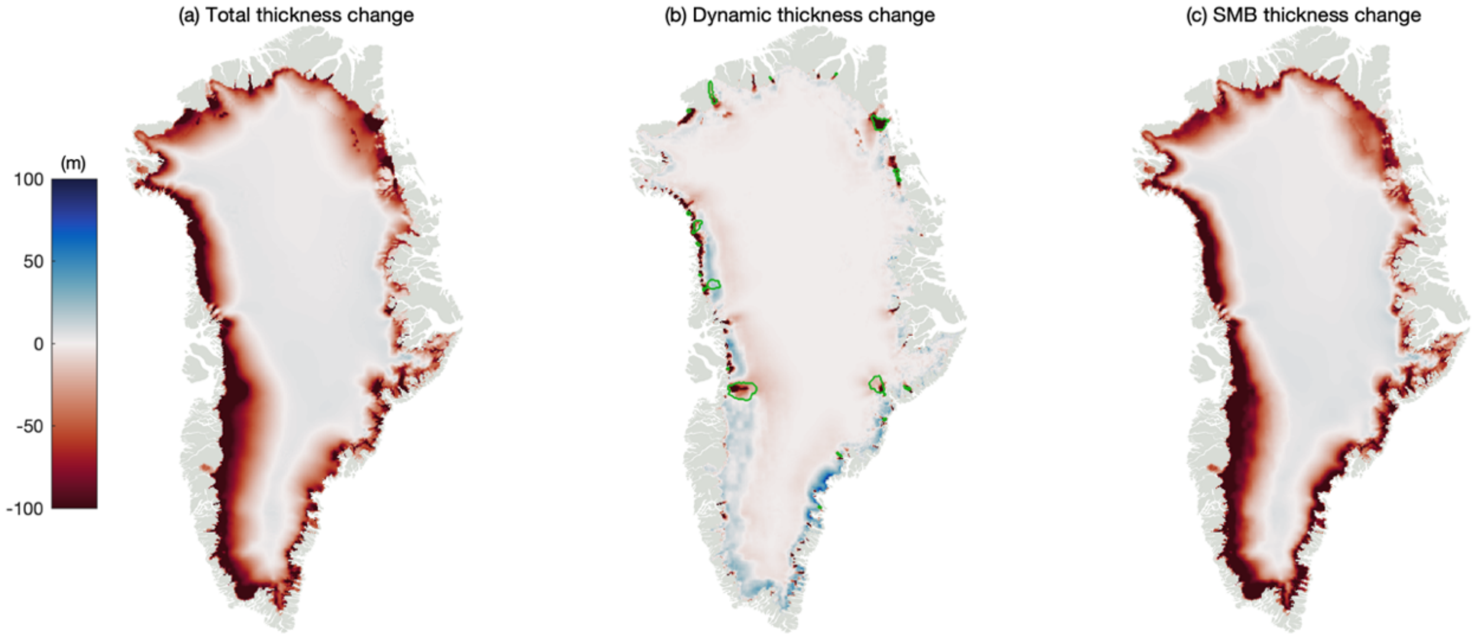

Supplementary Fig. 5. Simulated (a) total, (b) dynamic and (c) surface mass balance (SMB) elevation changes between 2015 and 2100 from an ensemble of ISMIP6 model experiments forced by the RCP8.5 emissions scenario. Contoured areas represent areas identified to be in a state of ice dynamical imbalance (green). Ice sheet projections taken from Goelzer et al.[72]

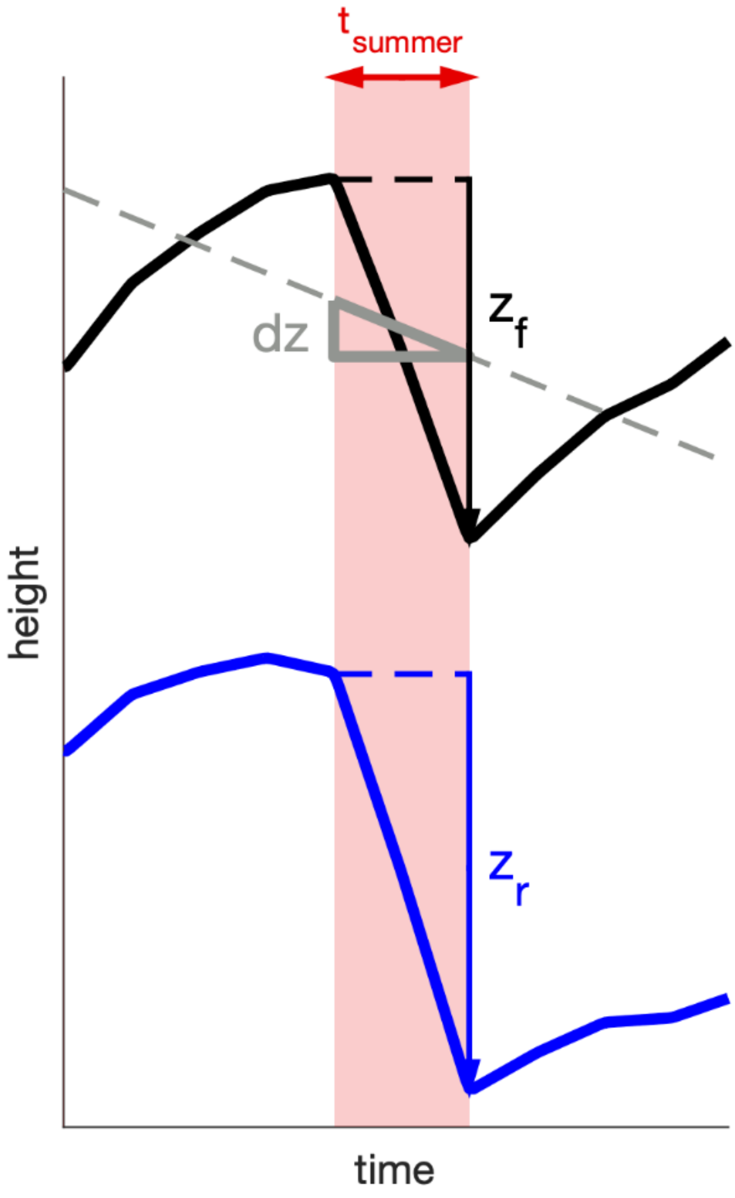

Supplementary Fig. 6. Schematic illustrating definitions used in equation (4). (top) The observed surface height change due to surface processes (z_f, black), which does not include the height change associated with the long-term mean advection (grey). (bottom) Height change due to runoff (z_r, blue) accounting for the surface height change in summer due to ice advection (dz).

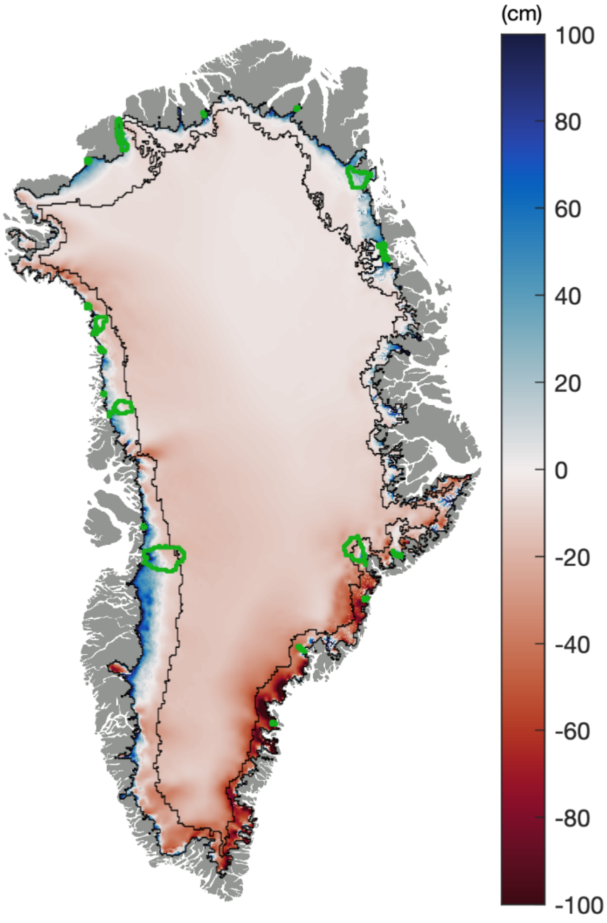

Supplementary Fig. 7. Map of the surface height change occurring over the summer melt season associated with ice advection ($dz \times \left(\frac{t_{summer}}{12}\right)$, equation (4)), estimated from the RACMO2.3p2 regional climate model and converted to height using a density of 917 kg/m^3 [20]. (black) Definition of the ice sheet ablation zone used in this study. We note that parts of the ablation zone near the inland boundary contain a negative surface height change: this is because the ablation zone is larger over our survey period (2011-2020) than when compared to period when the ice sheet was in steady state (1960-1980). (green) Definition of areas of long-term dynamical imbalance used in this study.

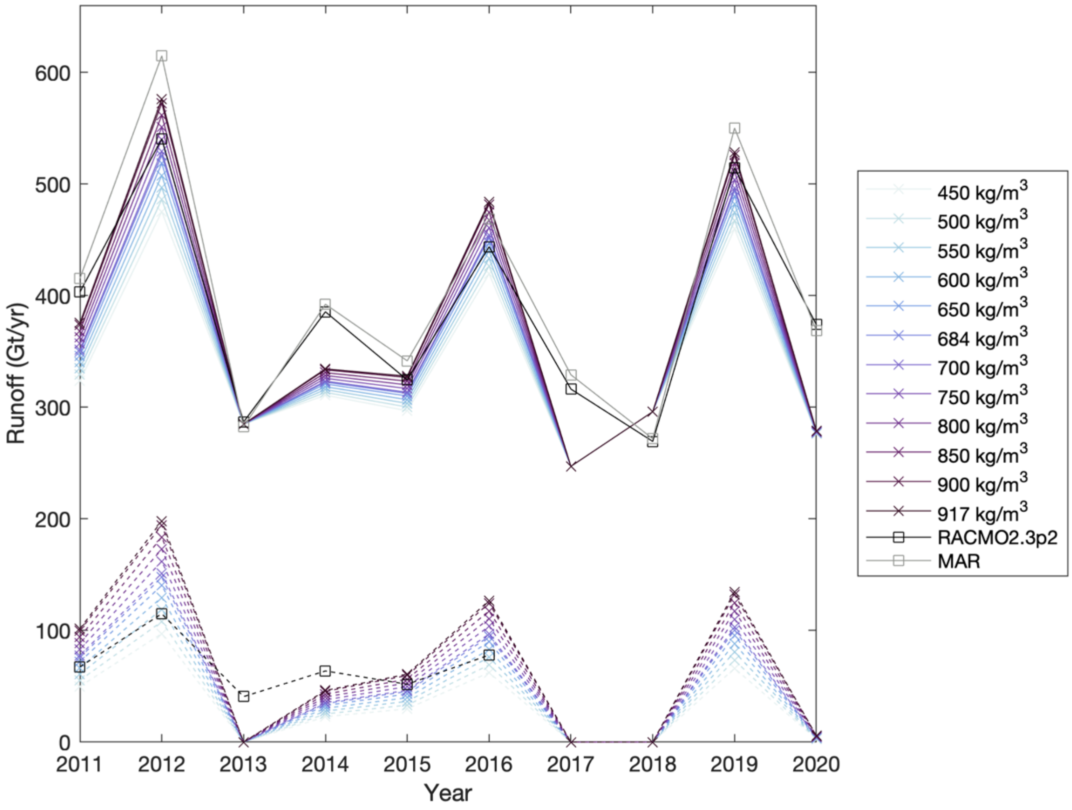

Supplementary Fig. 8. Runoff for the Greenland Ice Sheet (solid lines) and the region of the runoff zone inland from the ablation zone (dashed lines) derived from CryoSat-2 satellite observations, using different densities to convert elevation changes to runoff within the inland region. For the total runoff, a density of 917 kg/m^3 is used in the ablation zone. Runoff derived from (black) RACMO2.3p2 and (grey) MARv3.11 regional climate modelling is shown for comparison in each region, where it is available.

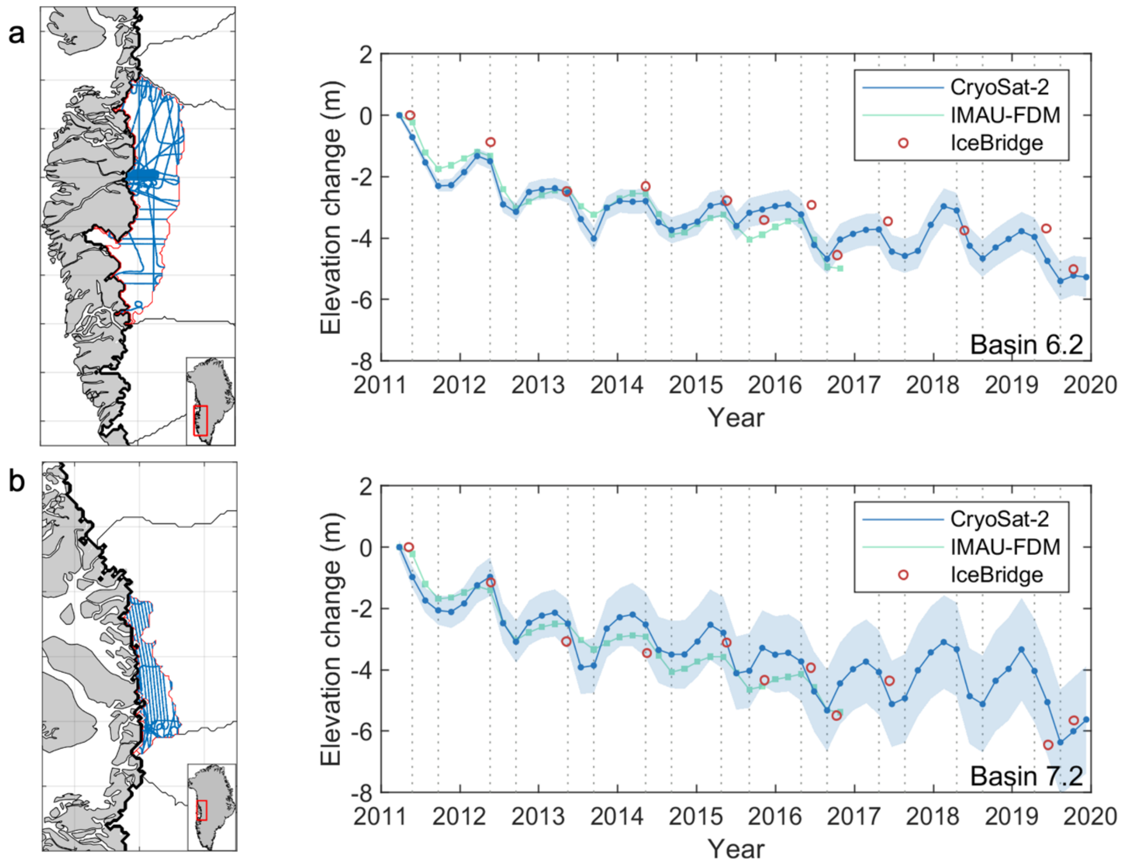

Supplementary Fig. 9. (Left) Locations of Operation Icebridge elevation data[38] used to compute time series of elevation change (right, red circles) for comparison with estimates derived from Cryosat-2 radar altimetry (dark blue) and from the Institute for Marine and Atmospheric Research Utrecht Firn Densification Model (IMAU-FDM) (light blue) in drainiage basins (a) 6.2 and (b) 7.2 (Fig. 2)[39].

	Dynamic elevation trend threshold (m/yr)									
	**0.1**	**0.2**	**0.3**	**0.4***	**0.5**	**0.6**	**0.7**	**0.8**	**0.9**	**1.0**
Area (km^2)	72800	42050	27625	20525	17250	14625	12925	10850	9000	8025
Runoff (2011-2020) (Gt/yr)	324	336	348	357	361	364	367	370	371	373
RACMO2.3p2 r.m.s.d (Gt/yr)	72	61	53	47	46	44	44	43	43	43
RACMO2.3p2 R^2	0.84	0.84	0.84	0.85	0.83	0.84	0.83	0.82	0.83	0.82
MARv3.11 r.m.s.d (Gt/yr)	90	78	68	60	58	55	54	52	52	51
MAR v3.11 R^2	0.87	0.87	0.88	0.88	0.87	0.87	0.86	0.86	0.86	0.86

Supplementary Table 1. Runoff estimates for the Greenland Ice Sheet using different dynamic elevation change rates to identify areas of dynamic imbalance to be excluded from the calculation.

* Choice of dynamic elevation trend threshold used in this study.

	Basin ID									
	**GrIS**	**1**	**2**	**3**	**4**	**5**	**6**	**7**	**8**	**Dynamic areas only**
SMB dh/dt (m/yr)	-0.86	-0.60	-0.56	-0.62	-0.95	-1.20	-1.20	-1.15	-1.03	-0.89
SMB thickness change (m)	-72.8	-51.0	-47.6	-52.3	-80.9	-102.2	-97.6	-96.3	-84.4	-76.0
Dynamics dh/dt (m/yr)	-0.03	-0.08	-0.10	0.01	0.14	0.14	0.07	-0.20	-0.23	-0.85
Dynamic thickness change (m)	-2.5	-6.5	-8.1	0.3	11.9	11.6	5.9	-16.9	-19.8	-72.0
Total dh/dt (m/yr)	-0.89	-0.68	-0.66	-0.61	-0.81	-1.07	-1.08	-1.33	-1.23	-1.74
Total thickness change (m)	-75.3	-57.5	-55.7	-52.0	-69.0	-90.6	-91.7	-113.2	-104.2	-148.0

Supplementary Table 2. Simulated average surface mass balance (SMB), dynamic and total elevation changes between 2015 and 2100 from an ensemble of ISMIP6 model experiments forced by the RCP8.5 emissions scenario. Thickness changes are presented for the runoff zone, with dynamic areas removed (i.e. the area our runoff estimates are calculated over) across the Greenland Ice Sheet and for the 8 drainage basins used in this study (Fig. 2), as well as within areas identified to be in a state of dynamical imbalance alone (Fig. 1). Ice sheet projections taken from Goelzer et al.[72]

Core ID	Publication	Latitude	Longitude	Average density of top 5 metres (kg/m³)
T1	Otosaka et al., 2020[56]	69.7310	48.1330	725.5
H4-1	Harper et al., 2012[55]	69.66018	48.68945	734.2
H5-1	Harper et al., 2012	69.64372	48.81594	836.7
T1-1	Harper et al., 2012	69.73802	48.06097	618.2
ACT_10_A	Box et al., 2013[54]	65.53	41.2	507.0

Supplementary Table 3. Firn cores used to determine the density used to estimate ice sheet runoff from CryoSat-2 satellite observations inland of the ablation zone.

			Inland density (kg/m³)											
		**Period**	**450**	**500**	**550**	**600**	**650**	**684***	**700**	**750**	**800**	**850**	**900**	**917**
**Total**	Runoff (Gt/yr)	2011-2020	339	343	346	350	354	357	357	361	365	368	372	373
**Total**	r.m.s.d (Gt/yr)	2011-2020	60	56	53	51	47	47	47	46	45	45	46	46
**Inland**	Runoff (Gt/yr)	2011-2020	33	37	40	44	48	50	51	55	59	63	66	67
**Inland**	r.m.s.d (Gt/yr)	2011-2016	28	25	23	23	24	26	27	30	34	39	44	46

Supplementary Table 4. Runoff estimates for the Greenland Ice Sheet (total) and the region of the runoff zone inland from the ablation zone from CryoSat-2 satellite observations, using different densities to convert elevation changes to runoff within the inland region. For the total runoff, a density of 917 kg/m³ is used in the ablation zone. Presented root mean squared differences are calculated with respect to RACMO2.3p2 estimates within the same region[20].

* Choice of density used in this study.
